# Neurocognitive function in males with 46,XX testicular difference of sex development

**DOI:** 10.1186/s13023-025-04126-z

**Published:** 2025-11-25

**Authors:** Etki Albayrak Rasborg Hartogsohn, Mirkka Hiort, Julia Rohayem, Jens Fedder, Sandra Laurentino, Jörg Gromoll, Silke Jörgens, Lukas Ochsner Reynaud Ridder, Anne Skakkebaek, Cecilie Buskbjerg, Agnethe Berglund, Claus Højbjerg Gravholt

**Affiliations:** 1 Department of Endocrinology, Aarhus University Hospital, Aarhus, Denmark; 2 Department of Molecular Medicine, Aarhus University Hospital, Aarhus, Denmark; 3 Department of Clinical Medicine, Aarhus University, Aarhus, Denmark; 4 Centre of Reproductive Medicine and Andrology, University of Münster, Münster, Germany; 5 Children’s Hospital of Eastern Switzerland, St. Gallen, Switzerland; 6 Centre of Andrology and Fertility Clinic, Odense University Hospital, Odense, Denmark; 7 Research Unit of Gynaecology and Obstetrics, University of Southern Denmark, Odense C, Denmark; 8 Institute of Reproductive Genetics, Centre of Medical Genetics, University of Münster, Münster, Germany; 9 Department of Psychiatry, University of Münster, Münster, Germany; 10 Department of Clinical Genetics, Aarhus University Hospital, Aarhus, Denmark; 11 Unit for Psychooncology and Health Psychology, Department of Psychology and Behavioral Sciences, Aarhus University, and Department of Oncology, Aarhus University Hospital, Aarhus, Denmark

## Abstract

**Background:**

46,XX testicular difference of sex development (46,XX T-DSD) is a rare condition, in which individuals with a typical female chromosomal pattern (46,XX) present with a male phenotype. Although neurocognitive function has previously been reported as normal in males with 46,XX T-DSD, studies indicate potential neurocognitive challenges, including lower educational attainment.

**Objective:**

We aimed to assess neurocognitive function in males with 46,XX T-DSD compared to 46,XY male controls using the Wechsler Adult Intelligence Scale, Fourth Edition (WAIS-IV).

**Methods:**

47 participants were included in the study, comprising 25 males with 46,XX T-DSD and 22 46,XY male controls matched on age and educational level. Of the 25 46,XX T-DSD males, 23 had an SRY translocation, while the remaining two were SRY-negative; one of these showed a SOX9 duplication, and no genetic cause was identified for the other despite extensive testing. All participants completed the WAIS-IV. We calculated each participant’s Full-Scale Intelligence Quotient (FSIQ) and four index scores: Verbal Comprehension Index, Perceptual Reasoning Index, Working Memory Index, and Processing Speed Index.

**Results:**

Males with 46,XX T-DSD scored significantly lower on the Working Memory Index (mean ± SD: 93.3 ± 15.7 vs. 104.3 ± 14.6, *p* = 0.017) compared to controls, with two of three subtests showing lower scores (*p* < 0.05). In the 46,XX T-DSD group, mean scores on the Verbal Comprehension Index and overall FSIQ were 91.6 ± 16.7 and 93.8 ± 15.6, respectively, compared with 98.9 ± 11.4 and 100.7 ± 10.3 in controls. Neither difference reached statistical significance (VCI: *p* = 0.092, FSIQ: *p* = 0.086). All mean scores for both groups remained within the normal range. Among males with 46,XX T-DSD, 56% (*n* = 14) scored in the low average range (80–89) or below on the FSIQ, compared to only 13.6% (*n* = 3) in the control group. Additionally, two males in the 46,XX T-DSD group scored in the extremely low range (≤ 69), whereas none in the control group did.

**Conclusion:**

Our findings indicate that 46,XX T-DSD males score significantly lower on the Working Memory Index compared to controls. No other statistically significant differences in index scores were observed, and all mean scores for both groups remained within the normal range. Larger-scale research and more comprehensive assessments of non-cognitive factors will be essential for gaining deeper insight into these findings and assessing their clinical significance.

## Introduction

46,XX difference of sex development (46,XX DSD, ORPHA:393) is a rare condition first described in 1964 by Albert de la Chapelle, where phenotypical males present with a typical female chromosomal pattern (46,XX) [1, 2]. The most common type of 46,XX DSD is testicular DSD (46,XX T-DSD), which affects approximately 2.7–3.8 per 100,000 newborn males, with a mean age at diagnosis of 25.4 years [1].

In 80–90% of 46,XX T-DSD cases, the cause is a translocation of the sex-determining region Y (SRY) gene from a Y chromosome to an X chromosome during paternal meiosis [1, 3, 4]. This leads to differentiation of the bipotential gonads into testes, which subsequently produce androgens and anti-Müllerian hormone (AMH), promoting virilization of the external genitals and regression of Müllerian structures [5]. In SRY-negative individuals, causes include small copy number variants in or near the SOX3 or SOX9 genes, as well as specific heterozygous pathogenic variants in NR5A1 or WT1 [6–9]. While the great majority of SRY-positive 46,XX T-DSD males develop fully mature male genitalia, most SRY-negative cases present with ambiguous genitalia [10].

46,XX T-DSD males exhibit a wide range of clinical characteristics, with the most prevalent being small testes, azoospermia, and hypergonadotropic hypogonadism, necessitating testosterone replacement therapy (TRT) [1, 11, 12]. Other frequently observed features include short stature, sparse body hair following a female-pattern distribution, gynecomastia, cryptorchidism, and hypospadias [12–14].

In other DSDs, such as Klinefelter syndrome (47,XXY), also characterized by hypergonadotropic hypogonadism and also necessitating TRT, the neurocognitive phenotype is well described [15, 16], whereas that of 46,XX T-DSD remains largely uncharacterized. While some studies have reported normal neurocognitive function [17], others have suggested impairments [18, 19]. In 1980, LaFranchi et al. described two adolescent boys with 46,XX DSD: one had learning difficulties, while the other demonstrated an intelligence quotient (IQ) of 85, which falls within the low-average range [18]. In a subsequent retrospective case series, the clinical features of 11 males with 46,XX DSD were reported, including four 46,XX T-DSD males, among whom two exhibited cognitive and/or learning impairments [19]. Additionally, a recent Danish registry study examined the socioeconomic status of 44 verified 46,XX DSD males, including 32 with 46,XX T-DSD, in comparison to 100 age-matched control males [1]. Education was defined by completion of a bachelor’s degree, and the study found that the 46,XX DSD group had a significantly lower rate of bachelor’s degree attainment compared to controls, with only one 46,XX DSD male achieving a bachelor’s degree [1]. Long-term income was also reduced in the 46,XX DSD group [1]. Similarly, a retrospective Chinese study collected educational data from 144 46,XX DSD males [13]. Based on the study’s criteria, T-DSD was defined by positive SRY status. SRY status was available for 86 of the 144 cases, and among these, 71 were SRY-positive. They found that 13.89% of the 144 46,XX DSD cases attained a bachelor’s degree, which was the highest level of education achieved within this cohort [13]. Neither of the retrospective studies distinguished between specific 46,XX DSD subgroups in their analysis of socioeconomic outcomes.

These findings suggest that 46,XX T-DSD males may exhibit some degree of neurocognitive challenges, as neurocognitive function is positively correlated with an individual’s ability to achieve higher academic performance and higher levels of education [20, 21]. To date, no study has systematically evaluated neurocognitive function in 46,XX T-DSD males, leaving a gap in our understanding of these patients’ neurocognitive profiles. This study seeks to investigate neurocognitive function in 46,XX T-DSD males compared to an age- and education-matched control group of 46,XY males.

## Materials and methods

### Participants

We recruited 25 males with a verified diagnosis of 46,XX T-DSD from the outpatient clinic at the Department of Endocrinology and Internal Medicine, Aarhus University Hospital, Denmark, and the Centre of Andrology and Fertility Clinic, Odense University Hospital, Denmark (*n* = 11), and the Centre of Reproductive Medicine and Andrology, University of Münster, Germany (*n* = 14). For the Danish cohort, 14 male controls were recruited. For the German cohort, 8 male controls were included from a previous study, in which exclusion criteria were self-reported prior thrombosis, current anticoagulation therapy or use of platelet inhibitors, current use of drugs of abuse, diabetes mellitus, and prior severe head trauma. No exclusion criteria were applied for the remaining cases or controls. Cases and controls were matched on educational level and age. For 23 of the 25 46,XX T-DSD males, the genetic background was an SRY-translocation, while the remaining two were SRY-negative. In one of these SRY-negative cases, we performed single nucleotide polymorphism array and identified a SOX9 duplication. In the other, extensive testing, including whole-genome sequencing, was performed, but no genetic cause was found, including duplications involving SOX3 or SOX9, pathogenic variants in NR5A1 or WT1, or other known causes of 46,XX T-DSD.

### Neurocognitive assessment

Neurocognitive function was evaluated using the Wechsler Adult Intelligence Scale – Fourth Edition (WAIS-IV), an internationally recognized and standardized test designed to assess adult intelligence and cognitive function [22]. It focuses on four primary cognitive indices: Verbal Comprehension Index (VCI), Perceptual Reasoning Index (PRI), Working Memory Index (WMI), and Processing Speed Index (PSI). Each index score is derived from a subset of 10 core subtests. Additionally, the WAIS-IV includes supplemental subtests, which can be used as substitutes for core subtests when necessary or to provide additional insights into specific cognitive functions. In the present study, four supplemental subtests were included to broaden the scope of the assessment. The included core and supplemental subtests are described in Table 1. The Digit Span subtest comprises three separate subtests: Digit Span Forward, Digit Span Backward, and Digit Span Sequencing.

**Table 1:** Overview of Wechsler adult intelligence Score – Fourth edition (WAIS-IV) indices and subtests

Index	Subtest	Evaluates
Verbal Comprehension Index (VCI)	Similarities	Verbal concept formation, abstract verbal reasoning
	Vocabulary	Verbal concept formation, language development
	Information	General knowledge, ability to acquire, store, and retrieve information
Perceptual Reasoning Index (PRI)	Block Design	Visual-motor coordination, abstract spatial reasoning, non-verbal concept formation and reasoning
	Matrix Reasoning	Fluid intelligence, abstract reasoning, categorization, visual-spatial problem-solving, simultaneous processing
	Visual Puzzles	Non-verbal reasoning, spatial reasoning, visual-spatial processing
	Figure Weights (S)	Quantitative reasoning, abstract reasoning, visual-spatial processing
	Picture Completion (S)	Visual perception and recognition
Working Memory Index (WMI)	Digit Span	Encoding, attention, concentration, mental manipulation, short-term memory
	Arithmetic	Mental calculation, attention, concentration, mental manipulation, mental alertness, short- and long-term memory
	Letter-Number Sequencing (S)	Mental manipulation, attention, concentration, sequential processing, memory span, auditory working memory
Processing Speed Index (PSI)	Symbol Search	Visual short-term memory, visual-motor coordination, visual discrimination, processing speed
	Coding	Visual short-term memory, psychomotor speed, visual scanning, concentration, attention, processing speed
	Cancellation (S)	Selective attention, visual discrimination, perceptual speed, concentration, attention, processing speed

(S) = Supplemental subtest

Raw scores from each subtest are converted into scaled scores using age-adjusted normative tables. Scaled scores have a mean of 10 and a standard deviation (SD) of 3. The scaled scores from relevant subtests are summed and converted into composite index scores (VCI, PRI, WMI, PSI), each with a mean of 100 and a SD of 15. The Full-Scale Intelligence Quotient (FSIQ), which provides an overall estimate of general intellectual ability, is derived by summing the scaled scores from the 10 core subtests and converting this sum using normative tables provided in the WAIS-IV scoring manual [22]. While the FSIQ reflects general cognitive ability, the index scores reflect specific cognitive strengths and weaknesses.

Table 2 categorizes FSIQ scores into standard classification ranges, from “Very superior” (130 and above) to “Extremely low” (69 and below) [22]. WAIS-IV testing was conducted by psychology master’s students under the supervision of an experienced clinical psychologist.

**Table 2:** Classification of Full-Scale IQ (FSIQ) scores from the Wechsler adult intelligence Scale–Fourth edition (WAIS-IV)

FSIQ score	Classification
130 and above	Very superior
120-129	Superior
110-119	High average
90-109	Average
80-89	Low average
70-79	Borderline
69 and below	Extremely low

### Statistical analysis

Statistical analyses were conducted using Stata (version 18.0, StataCorp LLC). Descriptive statistics (means ± SD for normally distributed variables and medians with 25th and 75th interquartile ranges (IQRs) for non-normally distributed variables) were calculated separately for the 46,XX T-DSD group and the control group. Comparisons between groups were made for the FSIQ, the four index scores, and the individual subtest scores. Independent samples t-tests were used for variables that met normality assumptions, whereas the Wilcoxon rank-sum test was employed as a non-parametric alternative whenever the assumptions of normality or homogeneity of variance were violated. Statistical significance was set at *p* < 0.05.

## Results

**Fig. 1:**
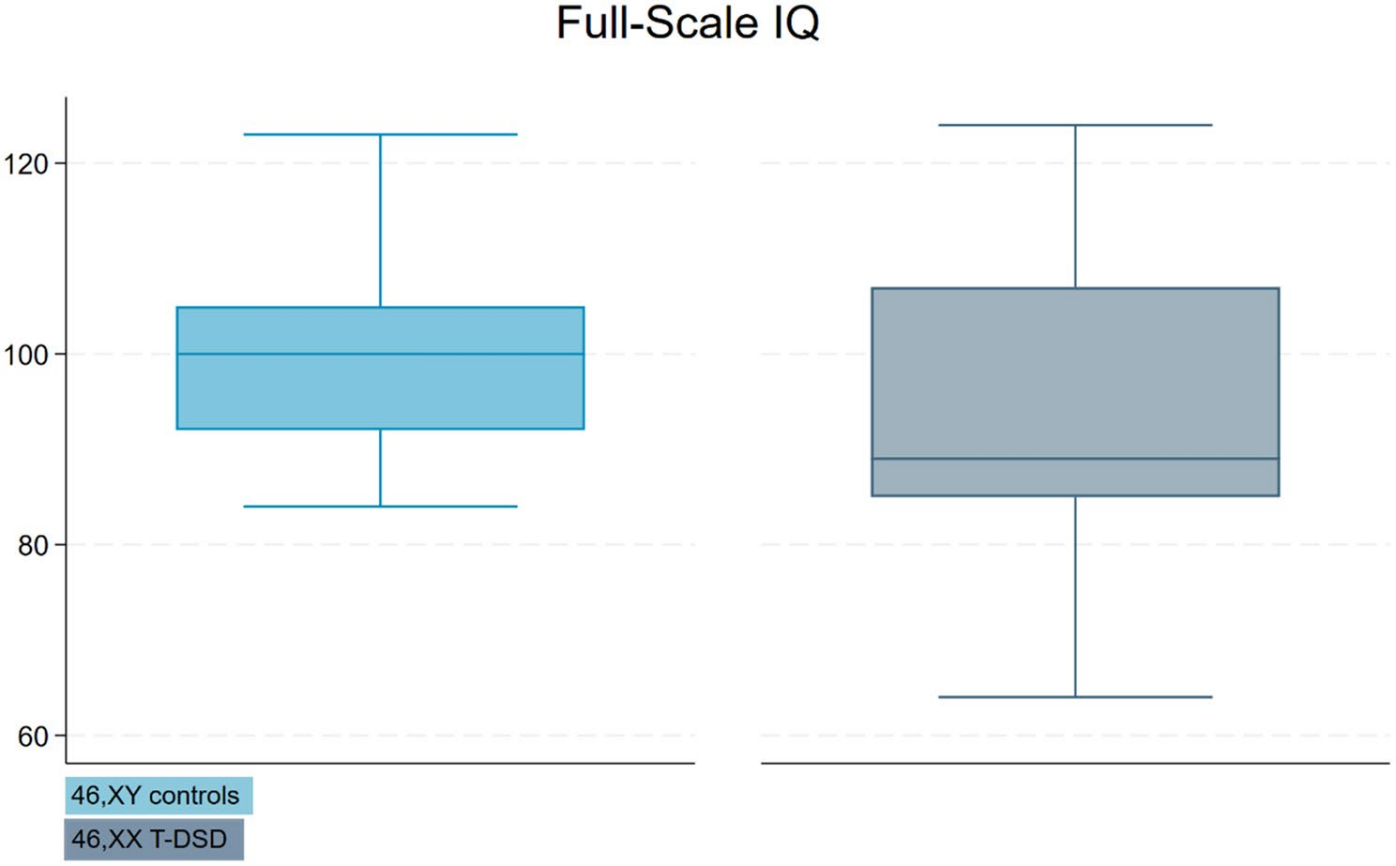
Boxplot of Full-Scale IQ Scores. Legend: 46,XY controls (light blue) and 46,XX T-DSD (gray). Each boxplot shows the median, interquartile range (IQR), and whiskers extending to 1.5 times the IQR. Outliers are represented as points outside the whiskers. Full-Scale IQ = Full-Scale Intelligence Quotient

**Fig. 2:**
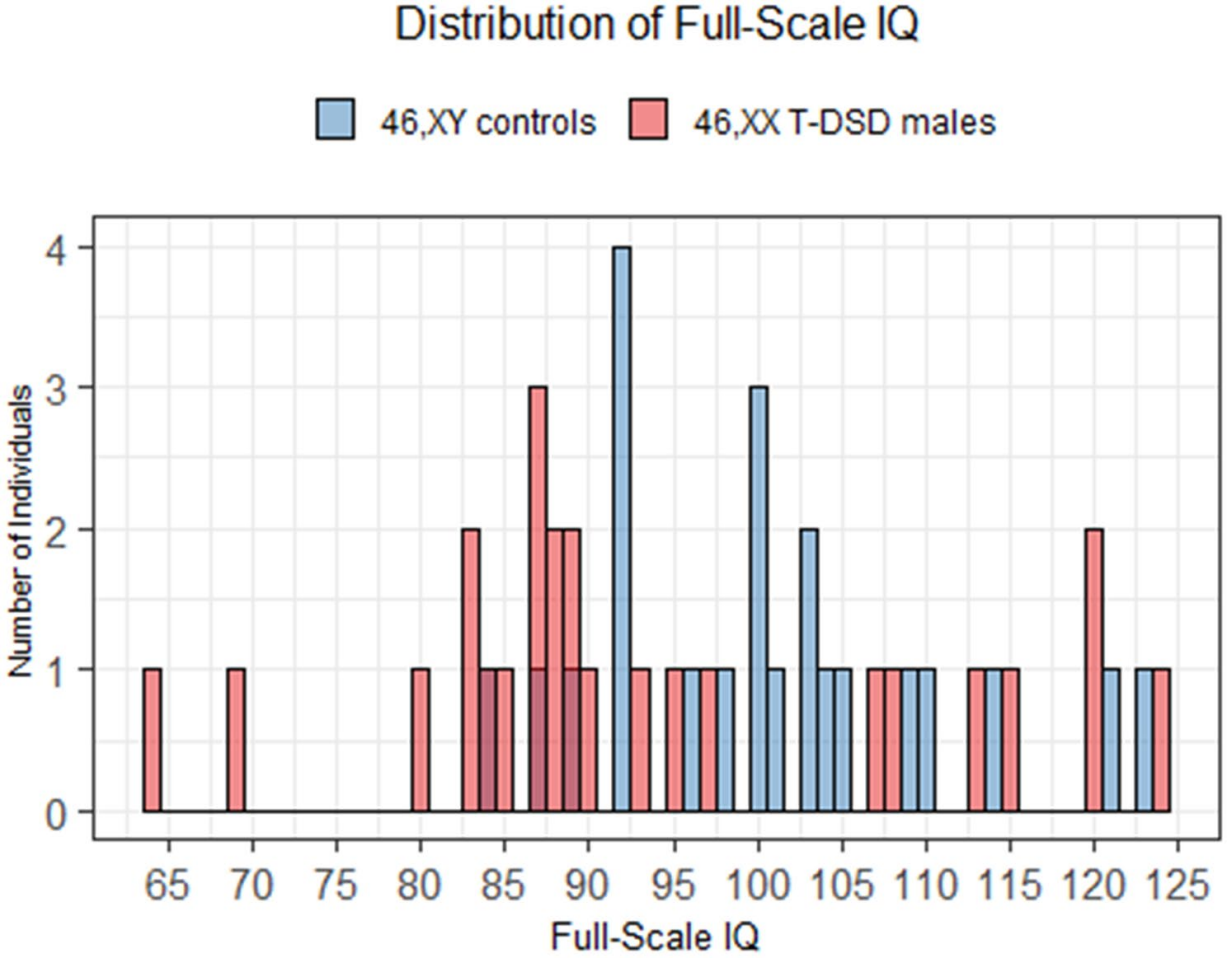
Histogram of Full-Scale IQ scores. Legend: 46,XY controls (blue) and 46,XX T-DSD males (red). Each bar represents the number of individuals with a given IQ score (1-point bins). Bars are semi-transparent and overlaid, highlighting group differences across the IQ range. Full-Scale IQ = Full-Scale Intelligence Quotient

The mean age of the 46,XX T-DSD males was 37.4 years (SD = 15.7), compared to 39.7 years (SD = 15.7) in the control group. The mean number of years of education was 12.3 (SD = 3.2) in the 46,XX T-DSD group and 12.7 (SD = 2.8) in the control group. There was no significant difference in age or length of education between the two groups (*p* = 0.6 and *p* = 0.7, respectively) (Table 3). Mean FSIQ score in the 46,XX T-DSD group was 93.8 ± 15.6 compared to 100.7 ± 10.3 in controls (Fig. 1), but this difference did not reach statistical significance (*p* = 0.086). In the 46,XX T-DSD group, 56% (*n* = 14) scored in the low average range or below on the FSIQ, compared to only 13.6% (*n* = 3) in the control group (Fig. 2). 8% (*n* = 2) of the 46,XX T-DSD males scored in the extremely low range, with scores of 64 and 69, respectively, and both were SRY-positive. None of the individuals in the control group had scores in the extremely low range. 20% (*n* = 5) of the 46,XX T-DSD males scored in the high average range or above, with three individuals in the superior range. In the control group, 18% (*n* = 4) scored in the high average range or above, with two scoring in the superior range.

**Table 3:** Summary of participant characteristics and scores on the Wechsler adult intelligence Scale–Fourth edition (WAIS-IV)

	46,XX T-DSD (*n* = 25)	46,XY (*n* = 22)	*P*-value
**Age (years)**	37.5 ± 15.7	39.7 ± 15.7	0.6
**Education (years)**	12.3 ± 3.2	12.7 ± 2.8	0.7
**Full-Scale IQ**	93.8 ± 15.6	100.7 ± 10.3	0.086
**Verbal Comprehension Index**	91.6 ± 16.7	98.9 ± 11.4	0.092
Similarities	7 (6-10)	10 (8-11)	**0.047**
Vocabulary	8.4 ± 3.3	8.6 ± 1.8	0.8
Information	8.2 ± 2.7	10.5 ± 2.6	**0.005**
**Perceptual Reasoning Index**	97.7 ± 17	101.8 ± 9.5	0.3
Block Design	10.1 ± 3.2	10.6 ± 1.8	0.5
Matrix Reasoning	9.1 ± 2.8	9.9 ± 2.4	0.3
Visual Puzzles	9.7 ± 3.4	10.4 ± 2.4	0.4
Figure Weights (S)	9.6 ± 3.2	11 ± 2.8	0.1
Picture Completion (S)	9.5 ± 2.1	10.2 ± 2.5	0.3
**Working Memory Index**	93.3 ± 15.7	104.3 ± 14.6	**0.017**
Digit Span	8.8 ± 3.1	10.5 ± 2.9	0.051
Arithmetic	9.1 ± 2.9	11.4 ± 3.1	**0.0098**
Letter-Number Sequencing (S)	9 (7-10)	10 (10-11)	**0.001**
**Processing Speed Index**	98.4 ± 12.7	99.1 ± 12.8	0.8
Symbol Search	9.7 ± 3.2	9.8 ± 2.8	0.9
Coding	9 (8-11)	9.5 (8-11)	0.9
Cancellation (S)	8 (7-10)	9 (8-13)	**0.037**

Full-Scale IQ and index scores are presented as standardized scores. Subtests are presented as scaled scores. Supplemental subtests are indicated by an (S). The results are presented as either means with standard deviations or medians with interquartile ranges, depending on normality. P-values are calculated using either a t-test or the Wilcoxon rank-sum test. Significant results are highlighted in bold

**Fig. 3:**
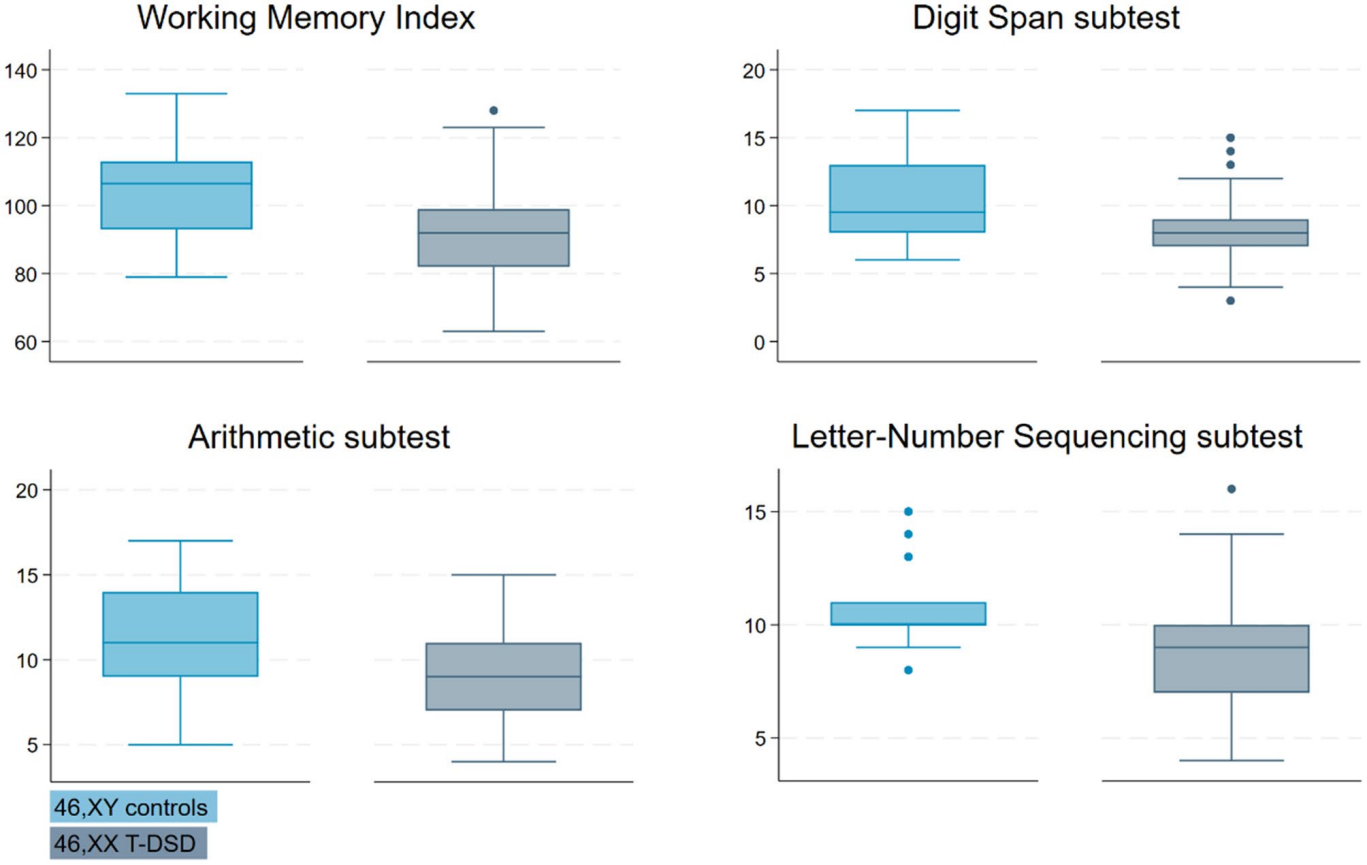
Boxplots of the Working Memory Index (WMI) and its subtests. Legend: 46,XY controls (light blue) and 46,XX T-DSD (gray). Each boxplot shows the median, interquartile range (IQR), and whiskers extending to 1.5 times the IQR. Outliers are represented as points outside the whiskers

46,XX T-DSD males had significantly lower scores on the WMI compared to controls (*p* = 0.017). They scored significantly lower in two of the three WMI subtests (Arithmetic and Letter-Number Sequencing), while the difference on Digit Span was not statistically significant (*p* = 0.051) (Fig. 3). Among the three components of the Digit Span subtest, the 46,XX T-DSD group had significantly lower scores on Digit Span Sequencing (*p* = 0.004), while no significant differences were observed in the Forward and Backward components. Digit Span subtest data were available for all controls but only for 11 of the 46,XX T-DSD males.

**Fig. 4:**
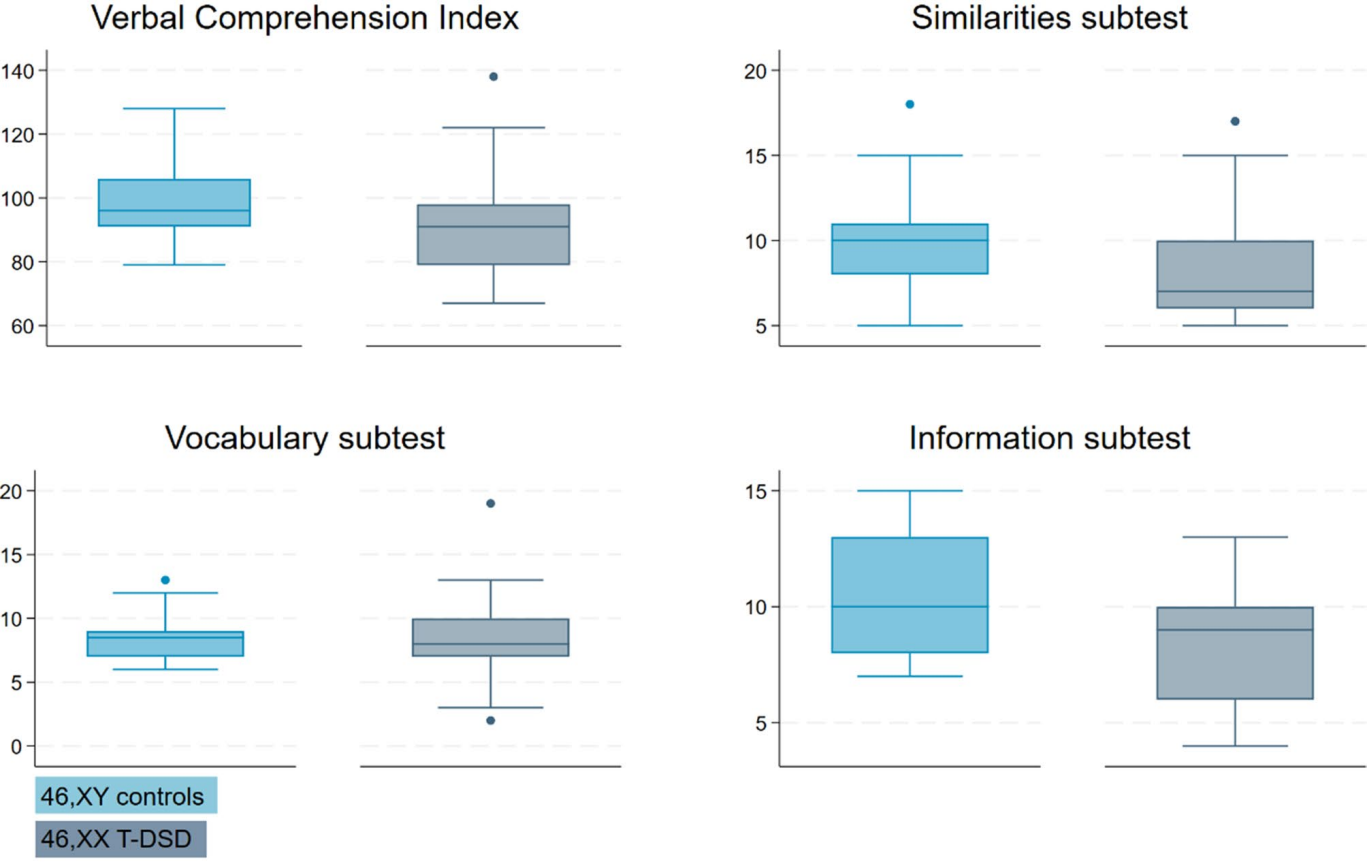
Boxplots of the Verbal Comprehension Index (VCI) and its subtests. Legend: 46,XY controls (light blue) and 46,XX T-DSD (gray). Each boxplot shows the median, interquartile range (IQR), and whiskers extending to 1.5 times the IQR. Outliers are represented as points outside the whiskers

**Fig. 5:**
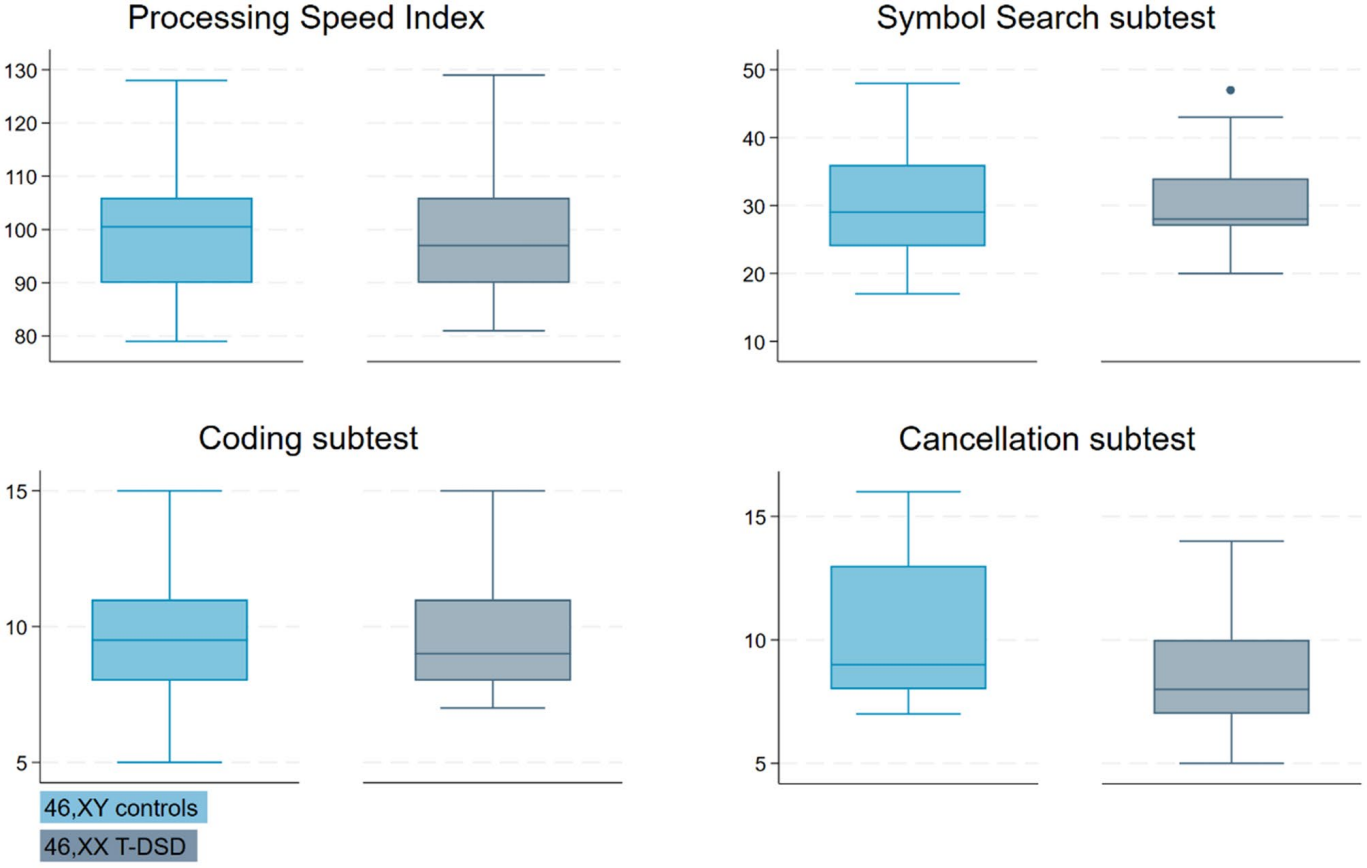
Boxplots of the Processing Speed Index (PSI) and its subtests. Legend: 46,XY controls (light blue) and 46,XX T-DSD (gray). Each boxplot shows the median, interquartile range (IQR), and whiskers extending to 1.5 times the IQR. Outliers are represented as points outside the whiskers

No significant differences were found in the other indices. However, significantly lower scores were observed on two of the three VCI subtests (Similarities and Information) in the 46,XX T-DSD group compared to controls (*p* = 0.047 and *p* = 0.005, respectively) (Fig. 4). Within the PSI subtests, 46,XX T-DSD males scored significantly lower on the Cancellation subtest compared to controls (*p* = 0.037) (Fig. 5).

## Discussion

This study represents, to the best of the authors’ knowledge, the first systematic evaluation of neurocognitive function in 46,XX T-DSD males compared to age- and education-matched 46,XY controls. Our findings indicate that, although 46,XX T-DSD males demonstrate significantly lower working memory scores compared to controls, their group mean remains within the normal range. Scores on the remaining indices were generally similar between groups, although a few subtest scores were significantly lower in the 46,XX T-DSD group. FSIQ did not differ significantly between the two groups, indicating that overall intellectual ability is not impaired in 46,XX T-DSD males.

Notably, more than half of the 46,XX T-DSD males scored in the low average range or below on the FSIQ, compared to only 13.6% of the controls. The two groups were more comparable at the upper end of the spectrum, leaving a larger proportion of male controls in the average range. This indicates that, although 46,XX T-DSD males and 46,XY controls are similarly represented at the higher FSIQ range, the overall distribution in 46,XX T-DSD males is nonetheless skewed more toward the lower end.

Neurocognitive difficulties are a well-established feature in some other DSDs, most notably KS, where deficits are observed across multiple domains, including language, executive function, and working memory [23]. Overall intelligence has also been shown to be significantly lower when compared to controls [16]. As in 46,XX T-DSD, KS is associated with significantly lower educational attainment and lower income, which is thought to be partially caused by these neurocognitive challenges [16, 24, 25]. While KS males exhibit broader neurocognitive deficits, males with 46,XX T-DSD in the present study exhibited a more specific pattern, with significantly lower scores on working memory that nevertheless remained within the normal range, while all other cognitive scores were comparable with controls and also within the normal range. This indicates that the neurocognitive phenotype of 46,XX T-DSD differs from that of KS, being more circumscribed and domain-specific rather than generalized. The neurocognitive deficits in KS are thought to be partially due to the presence of an extra X-chromosome, which may disrupt normal neurodevelopmental processes [15]. In contrast, 46,XX T-DSD does not involve an extra chromosome, but a common feature in both groups is hypergonadotropic hypogonadism in adulthood, resulting in testosterone deficiency [1, 26]. Males with 46,XX T-DSD and those with KS are typically prescribed TRT upon diagnosis to manage this condition [1]. However, diagnosis frequently occurs relatively late in both conditions [1, 27], and therefore, they may experience prolonged periods of testosterone deficiency. Testosterone plays an important role in neurocognitive processes, such as executive function, attention, and memory [28–30]. Therefore, this deficiency may contribute to some of the neurocognitive differences seen in both 46,XX T-DSD and KS.

Given the selective profile, the finding of significantly lower scores on working memory in 46,XX T-DSD warrants particular attention. Working memory plays a crucial role in learning, problem-solving, and daily functioning, serving as the cognitive system that temporarily holds and manipulates information [31, 32]. Impairments in this domain can limit an individual’s capacity to manage academic and occupational demands [31]. This is particularly relevant given previous studies showing that 46,XX T-DSD males attain significantly lower educational levels compared to controls [1, 13]. While the underlying causes are likely multifactorial, the present findings suggest that reduced working memory capacity may represent one contributing factor. Although scores in this domain remained within the normative range, mild weaknesses may still carry functional significance, especially when combined with environmental and psychosocial factors, such as motivation and self-esteem.

A key strength of this study is the inclusion of age- and education-matched controls, which enhances the accuracy of comparisons and reduces potential confounding related to developmental or academic differences. The use of the WAIS-IV, a comprehensive and internationally validated cognitive assessment tool, further strengthens the study by providing robust evaluation across multiple cognitive domains. However, it is important to note that the WAIS-IV does not capture other factors that may influence academic performance, such as motivation or personality traits, which should be addressed in future research [33, 34].

Despite these strengths, several limitations should be acknowledged. The small sample size may limit the generalizability of our findings. Additionally, p-values were not adjusted for multiple comparisons, and conducting numerous statistical tests increases the risk of type I errors. Therefore, findings approaching significance should be interpreted with caution. Another limitation is the fact that we could not identify the cause of 46,XX T-DSD in one patient despite performing extensive genetic testing. This is particularly relevant since phenotypic variability, including the neurocognitive profile, is known to be influenced by distinct underlying genetic mechanisms [3]. For example, duplications or pathogenetic variants including genes such as SOX3, SOX9, NR5A1, and WT1 have been associated with neurodevelopmental differences [8, 35–37]. Therefore, the absence of a confirmed genetic diagnosis in one patient limits our ability to interpret the neurocognitive findings in this specific case. Furthermore, since two males (both SRY-positive) scored extremely low on the FSIQ and fall within the range of intellectual disability, it would be valuable to investigate whether they carry gene variants associated with intellectual disability to clarify whether the deficits are due to the 46,XX T-DSD itself or from concomitant genetic anomalies.

Our findings suggest that 46,XX T-DSD males exhibit significantly lower working memory scores compared to controls, although their group mean score remains within the normal range. Future research with larger cohorts, genetic analyses, and more comprehensive neuropsychological assessments – including measures of non-cognitive factors – will be essential to further clarify these observations and determine their clinical significance.

## Data Availability

The datasets generated and analyzed during the current study are not publicly available due to restrictions related to patient confidentiality and ethical considerations. Access to the data may be available upon reasonable request and subject to approval by the relevant institutional review board and compliance with applicable legal regulations.
